# The PB2 mutation with lysine at 627 enhances the pathogenicity of avian influenza (H7N9) virus which belongs to a non-zoonotic lineage

**DOI:** 10.1038/s41598-017-02598-z

**Published:** 2017-05-24

**Authors:** Weixuan Li, Horace Hok Yeung Lee, Run Feng Li, Huachen Maria Zhu, Guan Yi, Joseph Sriyal Malik Peiris, Zi Feng Yang, Chris Ka Pun Mok

**Affiliations:** 1 Department of clinical laboratory, First people’s hospital of Foshan, Foshan, China; 2 HKU-Pasteur Research Pole, School of Public Health, HKU Li Ka Shing Faculty of Medicine, The University of Hong Kong, Hong Kong, China; 3 State Key Laboratory of Respiratory Disease, National Clinical Research Center for Respiratory Disease, First Affiliated Hospital of Guangzhou Medical University, Guangzhou, China; 4 Centre of Influenza Research, School of Public Health, HKU Li Ka Shing Faculty of Medicine, The University of Hong Kong, Hong Kong, China; 5 State Key Laboratory of Quality Research in Chinese Medicine, Macau University of Science and Technology, Macau, China

## Abstract

A novel avian-origin influenza A (H7N9) virus emerged in China in 2013 and has caused zoonotic disease in over 1123 persons with an overall mortality around 30%. Amino acid changes at the residues 591, 627 and 701 of polymerase basic protein 2 (PB2) have been found frequently in the human H7N9 isolates but not in viruses isolated from avian species. We have recently identified a cluster of H7N9 viruses in ducks which circulated in China prior to the first recognition of zoonotic disease in 2013. These duck viruses have genetic background distinct from the zoonotic H7N9 lineage. We found that the introduction of PB2 mutation with K at 627 but not K at 591 or N at 701 to the duck H7N9 virus led to increased pathogenicity in mice. We also found that the induction of pro-inflammatory cytokines including TNF-α, IP-10, MCP-1 and MIP-1α were associated with increased severity of infection. We conclude that introduction of the mammalian adaptation mutations into the PB2 gene of duck H7N9 viruses, which are genetically unrelated to the zoonotic H7N9 lineage, can also enhance pathogenicity in mice.

## Introduction

Zoonotic disease with avian-origin H7N9 influenza A (A/H7N9) viruses was identified in March 2013 leading to severe human disease and death in China. As of February 2017, there have been more than 1123 cases of human infections with at least 380 deaths^1^. Patients with the A/H7N9 disease typically developed a rapidly progressive viral pneumonia leading to respiratory failure and acute respiratory distress syndrome (ARDS) reminiscent of human HPAI H5N1 disease^2–5^. Although the pathogenic mechanism of this H7N9 subtype in human is still not clear, it has been suggested that, similar to the H5N1 virus, virus replication as well as cytokine dysregulation both contribute to disease severity. This hypothesis was further supported by studies using clinical specimens, human *ex vivo* cultures and *in vivo* mouse models^6–8^.

Human infections by other H7 avian subtypes including H7N3 and H7N7 have been reported previously in other countries^9, 10^. Prior to 2013, H7 subtype viruses have previously caused little impact in humans or poultry in China. We have recently elucidated the genesis and the origin of the H7N9 viruses causing zoonotic disease in China through the data obtained from our active surveillance in poultry and wild birds^11^. H7 influenza viruses from waterfowl from the East Asian migratory flyway were introduced into domestic ducks in China during the last decade and further reassorted with the local circulating viruses to generate different H7Nx virus subtypes. At least five H7Nx subtypes circulated in China during 2009-2010 and they were found to have distant genetic diversity. Interestingly, a cluster of avian H7N9 viruses circulated in ducks in Jiangxi during the period 2009-2011. On the other hand, a H7N3 virus further reassorted with another N9 subtype virus and then with H9N2 viruses from chicken (which donated the 6 internal genes) to form the new emerging zoonotic H7N9 lineage. This new lineage became established within poultry in China, causing the 2013 zoonotic disease outbreak.

The reason why infection with these zoonotic H7N9 viruses caused severe disease in humans is still not well understood. To overcome the restriction posed by the host barrier, avian viruses acquire mammalian adaptive mutations when they cross to mammalian hosts. In addition to the changes in the haemaglutinin gene (HA) which leads to the switching of binding affinity from Sia α2-3 Gal (receptor in avian host) to Sia α2-6 Gal (receptor in human host), adaptive mutations in the polymerase subunits are known to enhance the replication efficiency of avian influenza viruses in humans and other mammals^12^. PB2 mutations with K at 627, K at 591 and N at 701 have been identified as key viral determinants to enhance the pathogenicity of avian influenza virus in mammals^13–15^. These mutations were identified in the human H7N9 isolates and we have subsequently demonstrated their contribution on pathogenicity using both *ex vivo* human lung cultures and in mouse infection models *in vivo* ^13, 16^.

The six internal gene cassette of poultry H9N2 viruses has been found in other zoonotic avian influenza viruses including H5N1, H10N8 as well as H7N9^2, 17, 18^. Given that the virus adapted a mutation at the PB2, it is not known if the six internal genes from the poultry H9N2 viruses contribute to the pathogenicity of the new emerging H7N9 virus in human. We have previously isolated a H7N9 virus from a duck (A/duck/JX/3286/2009) which circulated in China around 2009. This virus showed no genetic relationship with the zoonotic H7N9 lineage and did not have any mammalian adaptation changes in PB2. In our previous study, we demonstrated that this virus caused mild pathogenicity in mice but the polymerase activity could be enhanced by a PB2 mutation with K at 627 in human cells^7, 13^. In this study, we further investigated the impact of introducing PB2 mutations of K at 627, K at 591 and N at 701 in duck H7N9 virus on pathogenicity in mice *in vivo*.

## Materials and Methods

### Viruses and cells

The avian H7N9 virus A/duck/JX/3286/2009 (3286/H7N9) was first isolated from a duck and was serially passaged in the allantoic cavity of embryonated eggs. Human embryonic kidney (293 T), Madin-Darby canine kidney (MDCK) and human epithelial A549 cells were maintained in Eagle’s minimal essential medium (MEM) containing 10% fetal calf serum and antibiotics.

### Plasmid construction

The plasmids of the individual gene segments of the influenza viruses A/Shanghai/2/2013 (Sh2/H7N9) and duck 3286/H7N9 were constructed as previously described^13^. In brief, the cDNAs of the viruses were first synthesized by reverse transcription of viral RNA with an oligonucleotide (Uni-12) complementary to the conserved 3′ end of the viral RNA. The cDNA was then amplified by PCR with gene-specific primers. The RT-PCR products of viral RNA segments were cloned into the plasmid pHW2000. The PB2 mutant construct containing specific mutations was produced by PCR amplification with the primers possessing the desired mutation.

### Generation of recombinant viruses

The full set of eight plasmids with or without the PB2 mutation cloned from the individual virus strain were transfected into a co-culture of MDCK and 293 T cells. Three days after transfection, the supernatant with the recombinant viruses that were generated were inoculated into embryonated eggs and incubated for three more days. The virus stock used for infection in subsequent experiments was titrated by plaque forming assays on MDCK cells.

### Luciferase assays of viral polymerase activity

293 T cells monolayers were transfected with 125 ng of luciferase reporter plasmid (pluci) and 12.5 ng internal control plasmid (phRL-CMV) together with the mix of PB2, PB1, PA and NP plasmids in quantities of 125, 125, 125 and 250 ng respectively. The transfected cells were incubated at 33 °C, 37 or 39 °C. After 24 hour incubation, the supernatant were discarded and the cell extracts were prepared in 100 ul of lysis buffer. The luciferase levels were assayed with Luciferase Assay System (Promega) and detected by luminometer.

### Cell infection

A549 cells were seeded at 1X10^5^ cells per well in 24-well tissue-culture plates. The cells were infected at a multiplicity of infection (MOI) of 0.01 for the analysis of virus replication. After one hour of virus adsorption, the virus inoculum was removed and the cells were washed with PBS and incubated in corresponded culture medium supplemented with 1 mg/L and 0.2 mg/L N-p-tosyl-L- phenylalaninechloromethyl ketone-treated trypsin (Sigma, St Louis, MO, USA) respectively. Samples of culture supernatants were collected for virus titration.

### Mice experiments

Specific pathogen free female BALB/c mice (6–8 weeks old) were infected intranasally with the virus in 25 ul volume and the mice monitored daily for weight loss. The mice were sacrificed at the indicated days post-infection for virological and cytokine assays. The lungs were isolated and homogenized in 1 ml PBS. The study protocol was carried out in strict accordance with the recommendations and was approved by the Committee on the Use of Live Animals in Teaching and Research of the University of Hong Kong (CULATR 3465–14).

### Quantitative analysis of cytokines

Protein expression levels of TNF-α, IP-10, MCP-1, MIP-1α, MCP-3, RANTES, IFN-α, GM-CSF and MIP-1β in the lung homogenates were quantitatively determined by flow cytometry–based immunoassay (Flowcytomix Multiplex, Bender MedSystems). In brief, the lung homogenates were collected at day 3 and day 6 post-infection. 25 ul of each sample was processed according to the manufacturer’s protocol. The amount of cytokine (pg/mL) in the samples were acquired on a BD LSRII (BD Bioscience) and was calculated by FlowCytomix Pro 2.3 software (Bender MedSystems).

### Virus titration

The amount of virus in the supernatants was titrated on MDCK cells and the titers were reported as tissue culture infectious dose units per 100 ul (TCID_50_/100 ul).

### Histopathology of the mice lung

Lung tissues from virus-infected mice were fixed in 10% neutral buffered formalin for at least 24 h before processing. The tissues were embedded in paraffin by standard tissue processing procedures, sections cut at 4 um and affixed on glass slides. Standard haematoxylin eosin staining was carried out.

### Statistical analysis

Statistical significance of differences between experimental groups was determined by using unpaired, parametric Student’s *t* test. Values of p < 0.05 were considered as significant.

### Biosafety

All procedures involving the H7N9 viruses were carried out in biosafety level 3 facility.

## Results

### Mutations at the PB2 gene of duck 3286/H7N9 virus enhance the polymerase activity

**Figure 1:**
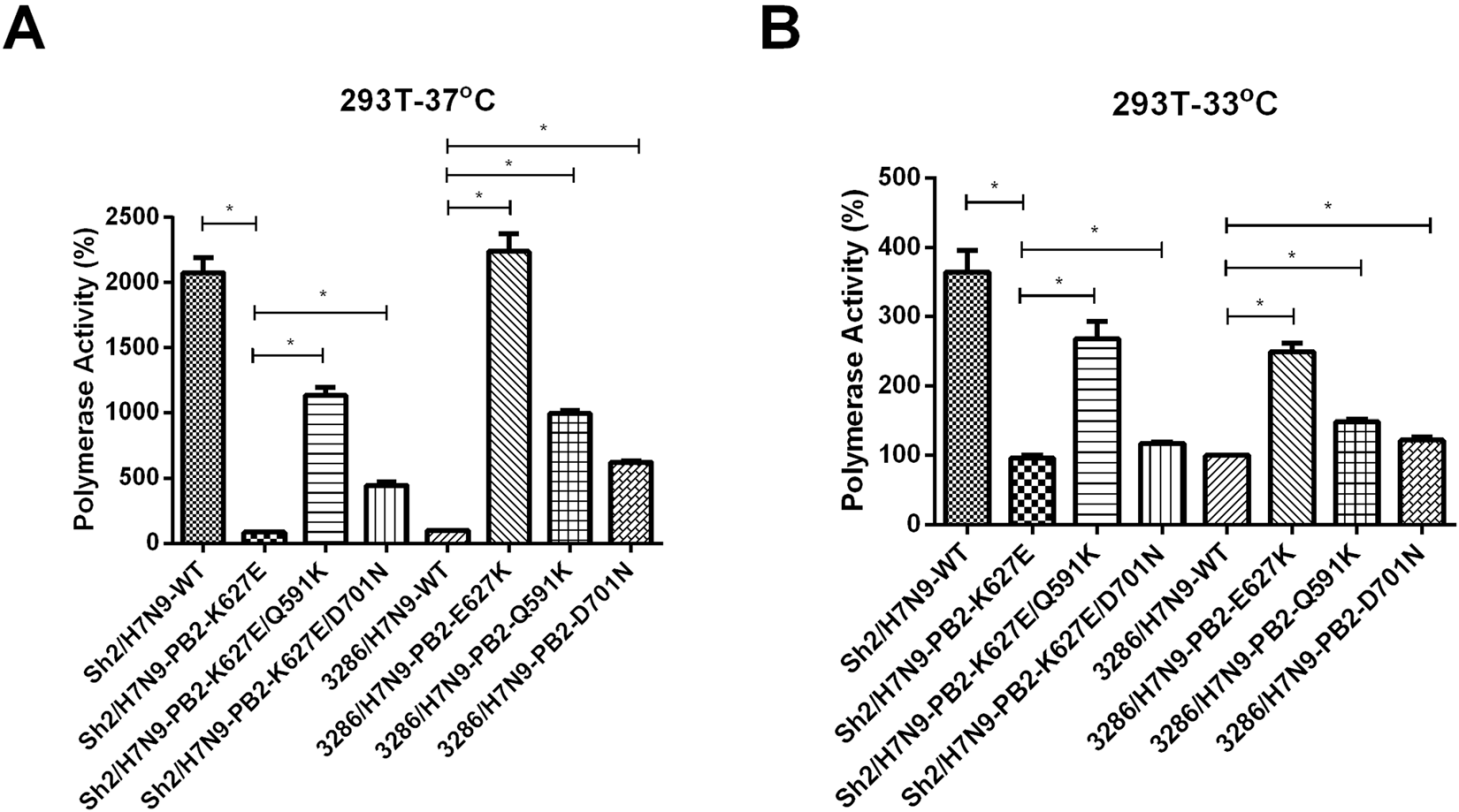
Polymerase activity of the H7N9 and the PB2 mutants. 293 T cells were transfected with plasmids containing PB2, PB1, PA, NP genes of either duck 3286/H7N9 or zoonotic Sh2/H7N9 plus a control luciferase reporter plasmid and a viral UTR-driven luciferase reporter plasmid. After transfection, the 293 T cells were cultured at (**A**) 37 °C and (**B**) 33 °C for 24 hour. Luciferase activity was then assayed from the cell extracts. Results are the average of three independent experiments. The values were statistical analyzed by two tailed, non-paired t-test. *p < 0.05.

We previously examined the PB2 gene sequences of the human H7N9 viruses and found that PB2 mammalian adaptations at the positions 591, 627 or 701 are frequently detected^7^. We also demonstrated that the mutations at these positions increased the polymerase activity of the viral ribo-nucleoprotein of a human H7N9 virus, A/Shanghai/2/2013 (Sh2/H7N9)^13^. When the PB2 mutation K at 627 was introduced in the duck 3286/H7N9 genome, a duck virus that belongs to a different avian lineage, we also observed enhanced polymerase activity in mammalian cells^13^. Here, we further compared the polymerase activity between the wild type duck 3286/H7N9 (627E) and the PB2 mutants with K at 591 and N at 701 by the mini-genome reporter assay using human 293 T cell line. Compared to the polymerase activity of the wild type PB2 627E, mutants with K at 627, K at 591 or N at 701 resulted in a higher polymerase activity at both 37 °C and 33 °C in mammalian cells (Fig. 1A and B) (p < 0.05). However, the increases in polymerase activity observed with K at 591 and N at 701 were less dramatic at 33 °C when compared to the results observed at 37 °C. As a positive control, similar effects on polymerase activity was observed when these PB2 mutations were introduced into the gene segments of the human H7N9 virus (Fig. 1A and B).

### Virus replication of the duck 3286/H7N9 virus in human cells is enhanced by PB2 mutations

**Figure 2:**
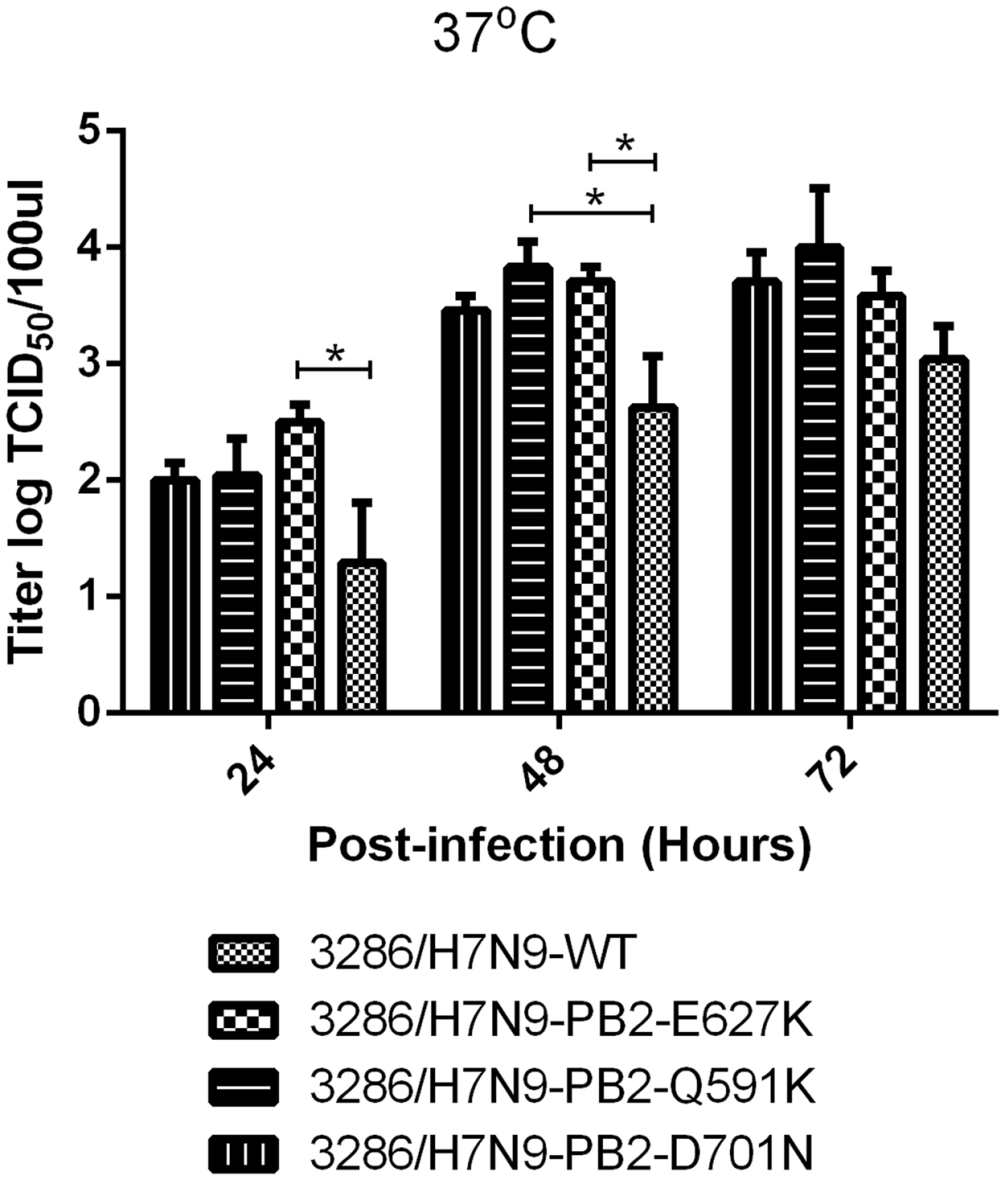
Replication kinetics of the duck 3286/H7N9 variants on A549 cells. A549 cells were infected with the indicated viruses at an MOI of 0.01 and cultured at 37 °C in the presence of TPCK-trypsin at 0.2 ug/ml respectively. Culture supernatants were harvested at the indicated times and virus titers were determined by TCID_50_ assay. Results are the average of three independent experiments. The viral titers in PB2 mutants were compared to the recombinant wild type virus using the two tailed, non-paired t-test. *p < 0.05.

We next examined the virus replication of the wild type duck 3286/H7N9 and its PB2 mutants in human lung epithelial cell line A549. Recombinant viruses of the wild type 627E and the PB2 mutants (K at 591, K at 627 and N at 701) were generated by virus reverse genetics. The A549 cells were infected with each recombinant virus at a multiplicity of infection (MOI) of 0.01 and were cultured at 37 °C. Supernatant from the infected cells was collected at 24-, 48-, 72-hr post-infection. Recombinant viruses with the PB2 mutation K at 627 replicated more efficiently than the wild type 627E virus in A549 cells at 24- and 48-hr post infection (Fig. 2). PB2 mutant with K at 591 showed higher virus replication than the wild type 627E virus at 48-hr post-infection while the PB2 mutant with N at 701 showed no difference to the wild type.

### The PB2 mutation with K at 627 in duck 3286/H7N9 virus contributes to increased pathogenicity in mice

**Figure 3:**
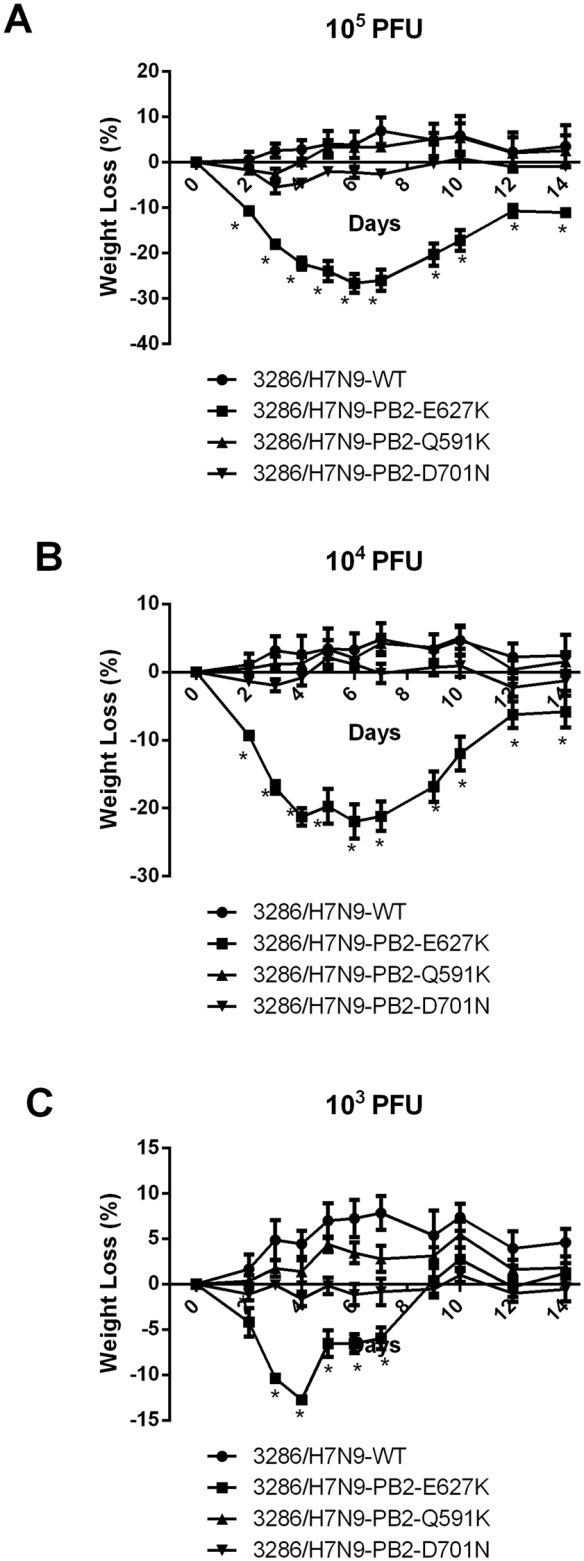
The weight change of the mice infected with the duck 3286/H7N9 and its PB2 mutants. Female BALB/c mice were infected intranasally with (**A**) 10^5^ PFU (**B**) 10^4^ PFU (**C**) 10^3^ PFU of the indicated viruses. The virus infected mice were monitored for 14 days and their weight were determined. Results from each group and each time point are expressed as mean ± SD of six infected mice from two independent experiments. The values were statistically analyzed by two tailed, non-paired t-test. *p < 0.05.

**Figure 4:**
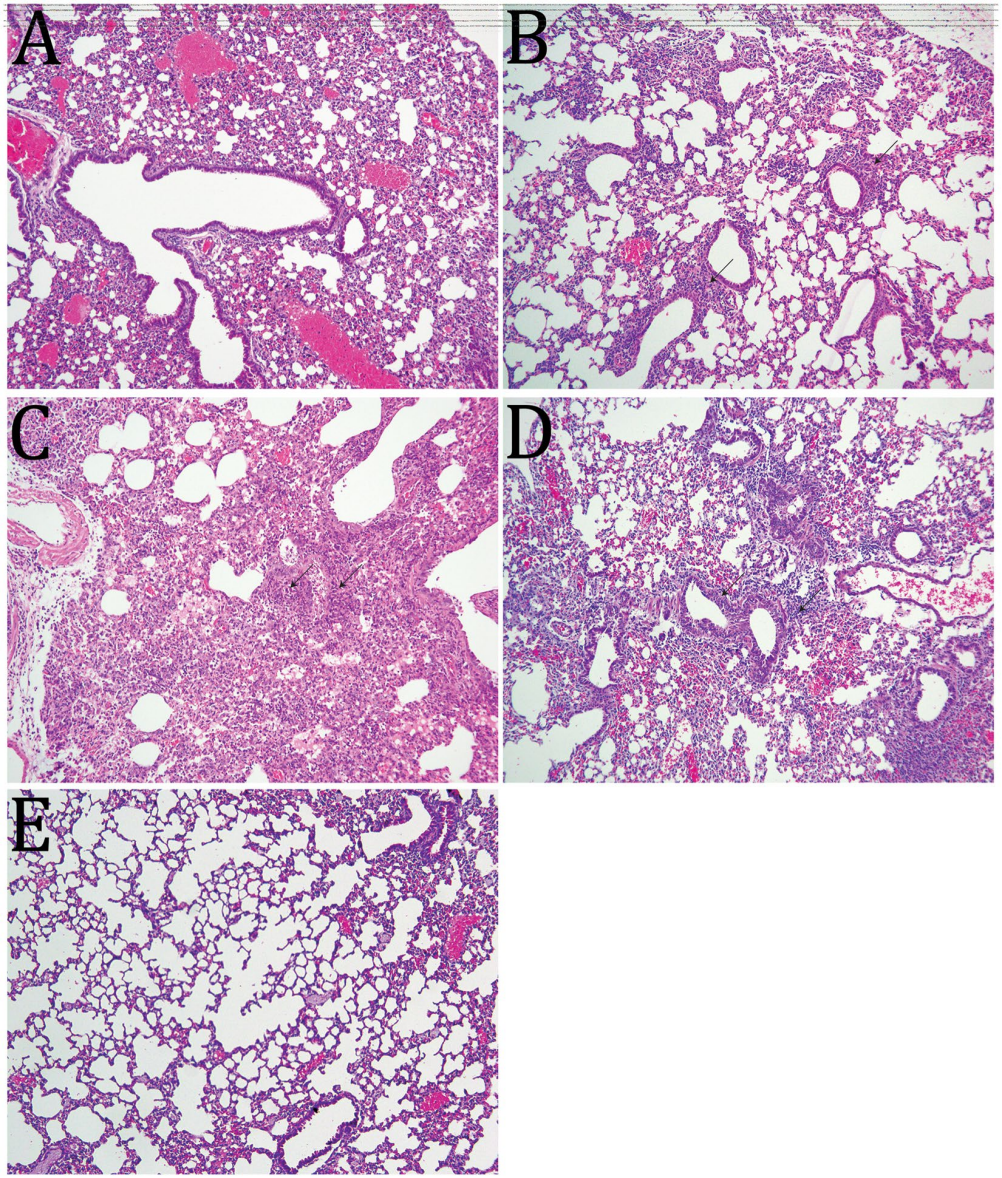
Histopathology of the mice infected with the duck 3286/H7N9 and its PB2 mutants. Histopathology of lung sections were determined from the samples stained by haematoxylin-eosin from mice infected with (**A**) duck 3286/H7N9, (**B**) 3286/H7N9-PB2- Q591K (**C**) 3286/H7N9-PB2-E627K (**D**) 3286/H7N9-PB2- D701N (**E**) Mock at 6 days post-infection. Magnification 100X. Arrow: Neutrophils infiltration.

**Figure 5:**
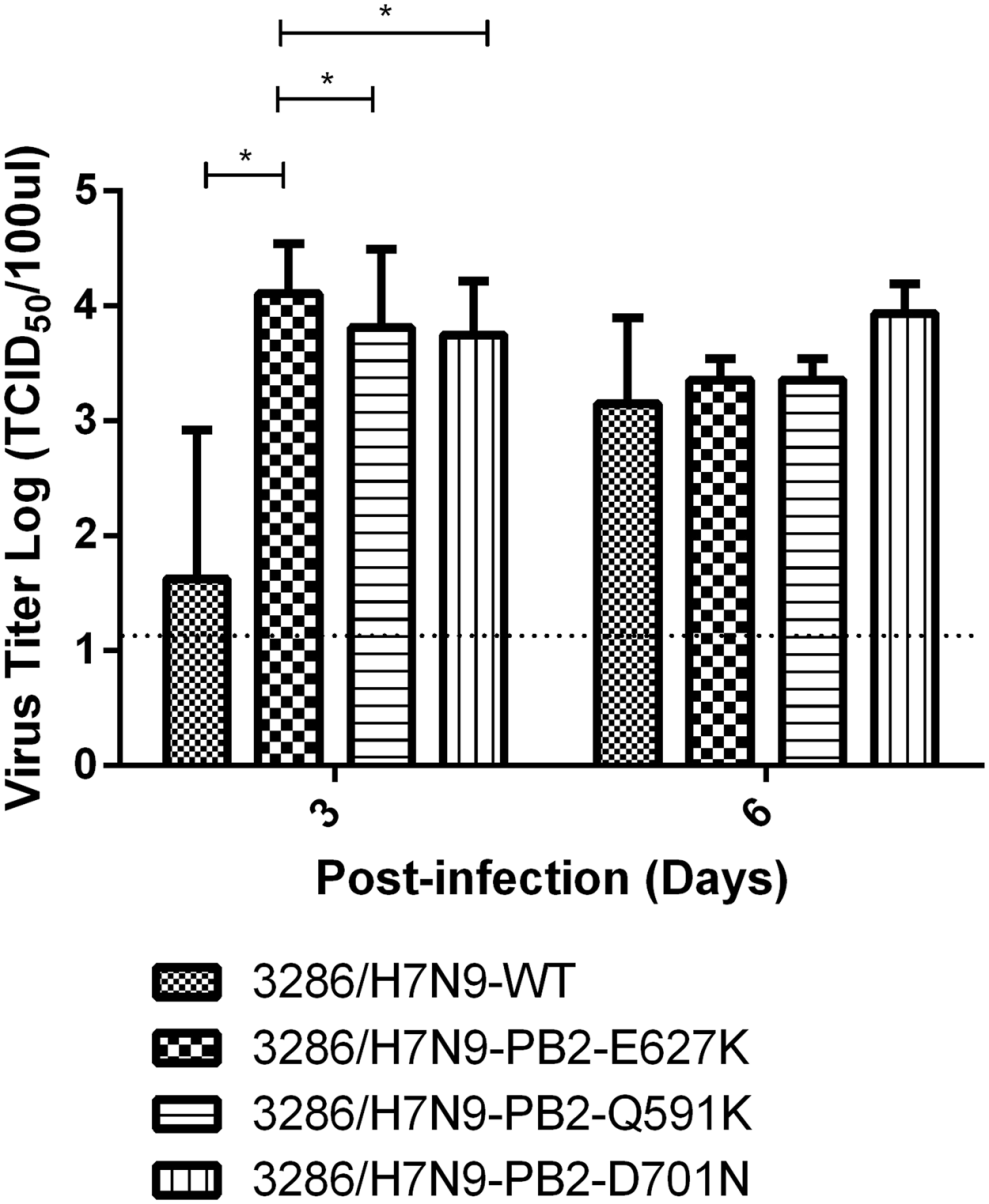
Lung virus titers of mice infected with duck 3286/H7N9 and its PB2 mutants. BALB/c mice were infected with the indicated viruses at 1 × 10^5^ PFU. Infected mice were sacrificed on 3 and 6 days post-infection, and virus titers in lung homogenates were measured in MDCK cells. Results from each group and each time point are expressed as mean ± SD of six infected mice from two independent experiments. The values were statistically compared with the wild type 3286/H7N9 using the two tailed, non-paired t-test. *p < 0.05.

To examine the pathogenicity of the recombinant viruses *in vivo*, we infected 6 to 8-week-old healthy female BALB/c mice with doses ranging from 1 × 10^3^ to 1 × 10^5^ PFU of the recombinant wild type 627E virus or its PB2 mutants intranasally. Mice inoculated with the viruses were monitored for 14 days and the weight loss and mortality were recorded (Fig. 3A–C). No mice died after infection of the wild type or recombinant viruses after 14 days of post-infection. Significant weight loss was observed in the mice infected by the PB2 mutant with K at 627 but they started to regain weight from 8 days after post-infection. No significant weight loss was observed in the mice infected by wild type 627E virus or PB2 mutants with K at 591 and N at 701. The extent of alveolar damage caused by the wild type and mutant viruses was assessed by histopathology (Fig. 4A–E). Overall, PB2 mutant with K at 627 caused the most severe pneumonia in the mice. The lungs of the mice infected by the wild type 627E, mutant with K at 591 or N at 701 showed only mild pathological change. In viruses with PB2 mutation K at 627, there was severe necrosis of bronchial epithelium, alveolar edema and severe multifocal alveolitis with infiltration of lymphocytes in inter-alveolar septa and a few neutrophils in alveolar lumen (Fig. 4C). In the mice infected by wild type 627E virus, tracheal dilatation and severe bronchiolitis with intra-epithelial lymphocytes was noticed. In addition, there was interstitial inflammation with cellular exudates containing lymphocytes and neutrophils, stenosis in the alveolar lumen, and congestion in alveolar capillaries (Fig. 4A). In the group of the PB2 mutation with K at 591, there was no apparent trachitis and mild alveolitis with infiltration of lymphocytes and lymphocytes (Fig. 4B). In the group of the PB2 mutation with N at 701, there was trachitis and bronchitis with few lymphocytes and plasmacytes. No inflammatory exudates were observed in alveolar lumen except a small amount of phagocytes. There was multifocal alveolitis with infiltration of lymphocytes in interalveolar septum (Fig. 4D). Mice with virus inoculated dose of 1 × 10^5^ PFU were sacrificed at day 3 and day 6 of post-infection. The virus replication and the cytokine induction in the lung were determined respectively. Mice infected by the PB2 mutants with K at 627, K at 591 and N at 701 showed higher virus titers (10^4^ TCID_50_/100 ul) compared to those infected with wild type 627E virus (10^2^-10^3^ TCID50/100 ul) at 3 days post infection (p < 0.05). There was no significant difference of virus titer between all groups at day 6 post-infection (p > 0.05) (Fig. 5). We did not detect any virus in the brain from the mice infected by the wild type or PB2 mutants (data not shown).

**Figure 6:**
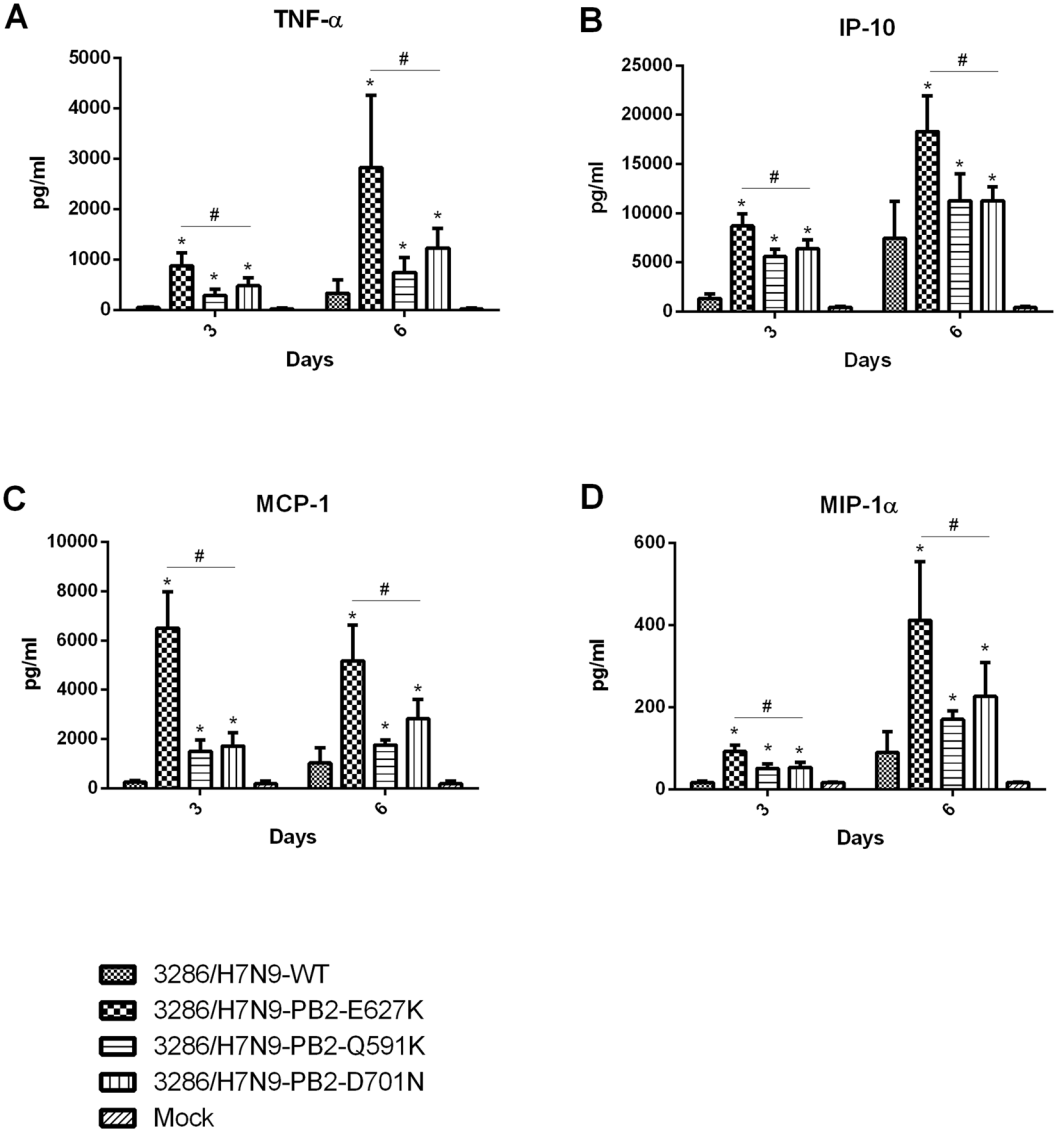
Cytokine responses in the lungs of mice infected with duck 3286/H7N9 and its PB2 mutants. Cytokine levels (**A**) TNF-α (**B**) IP-10 (**C**) MCP-1 (**D**) MIP-1α from virus infected lungs (n = 6 mice per virus group, days 3, 6 post-inoculation) were measured individually by the FlowCytomi*x* system. Results from each time point are expressed as mean ± SD of six infected mice from two independent experiments. *The values were statistically compared with the wild type 3286/H7N9 using the two tailed, non-paired t-test. p < 0.05. ^#^The values were statistically compared between the 3286/H7N9-PB2-E627K and other PB2 mutants using the two tailed, non-paired t-test. p < 0.05.

**Figure 7:**
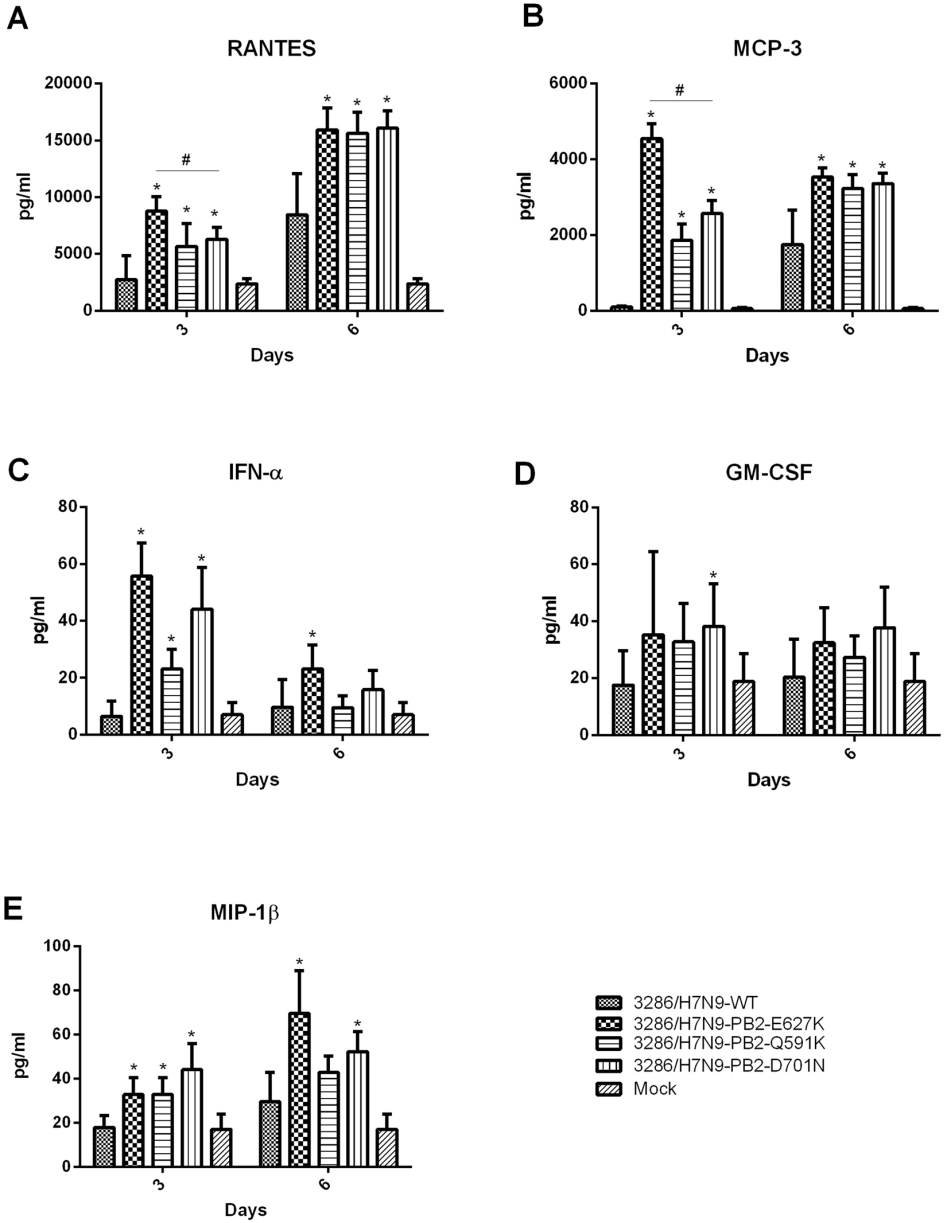
(cont) Cytokine responses in the lungs of mice infected with duck 3286/H7N9 and its PB2 mutants. Cytokine levels (**A**) RANTES (**B**) MCP-3 (**C**) IFN-α (**D**) GM-CSF E) MIP-1β from virus infected lungs (n = 6 mice per virus group, days 3, 6 post-inoculation) were measured individually by the FlowCytomi*x* system. Results from each time point are expressed as mean ± SD of six infected mice from two independent experiments. *The values were statistically compared with the wild type 3286/H7N9 using the two tailed, non-paired t-test. p < 0.05.

We have previously shown that PB2 mutations in the H7N9 human isolate enhanced the cytokine induction in the mice compared to the virus which does not have PB2 mutation at 591, 627 or 701. We now determined whether these PB2 mutations in duck 3286/H7N9 virus can also enhance the induction of pro-inflammatory cytokines in mice. Mice infected by the wild type 627E and the PB2 mutants all induced increased levels of pro-inflammatory cytokines (TNF-α, IP-10, MCP-1, MIP-1α, RANTES, MCP-3, IFN-α, MIP-1β and GM-CSF) in the lung compared to the uninfected control at both 3 and 6 days post-infection although the wild type 627E and PB2 mutants with K at 591 and N at 701 showed no significant weight loss (Figs 6 and 7). Among all recombinant viruses, we found that the PB2 mutant with K at 627 induced higher level of TNF-α, IP-10, MCP-1 and MIP-1α at both 3 and 6 days post-infection when compared to the wild type 627E virus and the other two PB2 mutants (Fig. 6). It is therefore possible that the increased cytokine induction may correlate with the pathogenicity in virus infected mice although what we have observed is an association rather than evidence of causation.

## Discussion

The new emerging zoonotic H7N9 lineage has been endemic in China since 2013, has spread to multiple provinces and has caused five waves of zoonotic disease^1, 19^. Phylogenetic analysis suggested that this human H7N9 lineage acquired six internal genes from avian H9N2 through natural reassortment, as occurred with zoonotic H5N1 and H10N8 viruses^2, 11^. However, whether the genetic background of this H7N9 lineage contributed to the pathogenicity in human is still not well understood. We previously obtained a duck H7N9 virus (3286/H7N9) which circulated in China around 2009 which has little genetic relationship to the zoonotic H7N9 lineage^11^. We also showed that this avian H7N9 virus which does not have any mammalian adaptation signatures at PB2, only cause mild disease in mice^7^. In this study, we aimed to further investigate the role of the PB2 mutation on the virulence of this duck H7N9 virus in mice. We showed that the PB2 mutant with K at 627 caused a dramatic weight loss in the mice which is similar to the results of using the virus from the zoonotic H7N9 lineage. Our results suggest that the genetic background of the human H7N9 lineage may not be the key factor to determine its pathogenicity in mammalian host and that the PB2 mutations are likely to be more important in this regard.

The PB2 mutation with K at 627 has been commonly found in human seasonal influenza strains as well as the human pathogenic H5N1, H7N9 and H10N8 viruses^2, 17, 18^. Most of the H5N1 influenza viruses which were isolated from avian hosts possess 627E at PB2 (with the exception of clade 2.2 strains) while 627 K at this position was frequently found in the H5N1 human isolates^20, 21^. Moreover, the 1918 H1N1 and the H7N7 viruses which were isolated from fatal cases also had the mutation with K at this position^22, 23^. It was suggested that high viral replication and cytokine dysregulation are the key determinants for the pathogenicity of avian viruses in humans^24^. Experimental studies showed that these factors were associated with this mutation^25, 26^. Our study demonstrated that the PB2 mutation with K at 627 in PB2 from zoonotic or duck H7N9 viruses significantly increased the polymerase activity in human cells at both 33 °C and 37 °C, which represent the temperatures at the upper and lower lung respectively. Mice infected by the recombinant virus with this mutation showed higher virus replication and cytokine induction in the lung compared to the wild type 627E virus. Similar results were also observed from our previous studies using H9N2 viruses that the 627 K mutation determined the virus replication as well as the level of inflammation in the mice^27^.

Although the PB2 mutation with K at 627 appeared to be the most frequent among all adaptive mutations, amino acid changes at other residues of the PB2 such as 591 K and 701 N have been observed and have been suggested to play a similar role as PB2 627 K given that the avian virus maintain its avian 627E signature at PB2^28, 29^. During the first wave of zoonotic H7N9 disease in China, viruses with PB2 mutations with K at 591 and N at 701 were also identified from the human isolates. Our results of the polymerase activity from the two H7N9 lineages showed that both mutations enhanced the viral transcriptional/translational efficiency at 37 °C. However, while the PB2 mutation with K at 591 but not N at 701 from the human H7N9 virus still dramatically enhanced the polymerase activity at 33 °C, in the duck H7N9 PB2 we used in this study, these mutations only slightly increased the polymerase activity. Studies showed that the two mutations only partially substituted the role of the mutation with K at 627 as demonstrated in human cells or pathogenicity in mice^13, 27, 30, 31^. In addition, it is noted that there are six additional amino acid differences between the PB2 of the human and duck H7N9 viruses. Whether they may contribute to the difference of the results needs to be further investigated. Surprisingly, the duck H7N9 virus with the mutation K at 591 or N at 701 showed comparable virulence to the wild type 627E virus with marginal weight loss (p > 0.05) being observed. Our previous results demonstrated that the recombinant H7N9 or H9N2 viruses that possess these two mutations also enhanced the pathogenicity in mice compared with the isogenic 627E virus although the phenotype was less pathogenic than the one with K at 627^13, 27^. The overall lung titres infected by the PB2 mutants were two logs higher in the H9N2 study than the mice infected by both lineages of H7N9 implicating that the high viral load in this case may in fact contribute to the pathogenicity. In H7N9, the level of the virus replication in the lungs did not correlate to the severity caused by the different PB2 mutants from both duck and zoonotic lineages. While the virus replication showed a compatible level at day 3, there was a significant difference among the three PB2 mutants (591, 627 and 701) later in the infection. It seem entirely possible from the results of the differences in replication *in vitro* that there are differences in replication for the 627 K group in mice earlier, on day 1 or day 2 when weight loss difference was already apparent.

Higher level of pro-inflammatory cytokines were found in the serum samples collected from the acute phase of patients infected by H7N9 or H5N1 compared to those collected from normal controls or in those infected with seasonal influenza viruses^8, 32^. These results strongly suggest that cytokines dysregulation contributes to the disease severity during the H7N9 infection. In this study, mice infected by the PB2 mutant with K at 627 induced higher amount of TNF-α, IP-10, MCP-1, MCP-3 and MIP-1α compared to the wild type 627E and PB2 mutants at 591 and 701. In particular, the levels of MCP-1 and -3 induced by the mutants with K at 591 and N at 701 were as low as the level observed at the wild type 627E virus. While these two cytokines were shown to associate with the pathogenicity of H5N1 and H7N9 in clinical studies, it may partly explain why these two PB2 mutants did not cause severe weight loss in the mice.

In conclusion, our study investigated and compared the role of the PB2 mammalian adaptation in two different H7N9 lineages using *in vitro* and *in vivo* models. These findings may help to further understand the mechanism of pathogenesis of the H7N9 subtype in human.

## References

[1] World Heath Organization. http://www.who.int/influenza/human_animal_interface /HAI_Risk_Assessment/en/ (2017).

[2] Gao R (2013). Human Infection with a Novel Avian-Origin Influenza A (H7N9) Virus. N Engl J Med..

[3] Yu L (2013). Clinical, virological and histopathological manifestations of fatal human infections by avian influenza A(H7N9) virus. Clin Infect Dis..

[4] Gao HN (2013). Clinical findings in 111 cases of influenza A (H7N9) virus infection. N Engl J Med.

[5] Wu P (2016). Human Infection with Influenza A(H7N9) Virus during 3 Major Epidemic Waves, China, 2013–2015. Emerg Infect Dis..

[6] Chan MCW (2013). Tropism and innate host responses of a novel avian influenza A H7N9 virus: an analysis of *ex-vivo* and *in-vitro* cultures of the human respiratory tract. The Lancet Respiratory Medicine..

[7] Mok, C.K. *et al*. Pathogenicity of the novel A/H7N9 influenza virus in mice. MBio. e00362-13 (2013). 10.1128/mBio.00362-13 PMC3705449 23820393

[8] Chi Y (2013). Cytokine and Chemokine Levels in Patients Infected With the Novel Avian Influenza A (H7N9) Virus in China. J Infect Dis..

[9] Belser JA (2013). Pathogenesis, transmissibility, and ocular tropism of a highly pathogenic avian influenza A (H7N3) virus associated with human conjunctivitis. J Virol..

[10] Koopmans M (2004). Transmission of H7N7 avian influenza A virus to human beings during a large outbreak in commercial poultry farms in the Netherlands. Lancet..

[11] Lam TT (2013). The genesis and source of the H7N9 influenza viruses causing human infections in China. Nature..

[12] Mänz, B., Schwemmle, M., Brunotte, L. Adaptation of avian influenza A virus polymerase in mammals to overcome the host species barrier. *J Virol*. 7:7200-9. Review (2013). 10.1128/JVI.00980-13 PMC3700283 23616660

[13] Mok CK (2014). Amino acid substitutions in polymerase basic protein 2 gene contribute to the pathogenicity of the novel A/H7N9 influenza virus in mammalian hosts. Journal of virology.

[14] Yamayoshi S (2015). Amino acids substitutions in the PB2 protein of H7N9 influenza A viruses are important for virulence in mammalian hosts. Scientific reports.

[15] Zhang H (2014). The PB2 E627K mutation contributes to the high polymerase activity and enhanced replication of H7N9 influenza virus. The Journal of general virology.

[16] Chan LL (2016). Evaluation of the human adaptation of influenza A/H7N9 virus in PB2 protein using human and swine respiratory tract explant cultures. Sci Rep..

[17] Chen H (2014). Clinical and epidemiological characteristics of a fatal case of avian influenza A H10N8 virus infection: a descriptive study. Lancet..

[18] Guan Y, Shortridge KF, Krauss S, Webster RG (1999). Molecular characterization of H9N2 influenza viruses: were they the donors of the “internal” genes of H5N1 viruses in Hong Kong?. Proc Natl Acad Sci USA.

[19] Xiang N (2016). Comparison of the first three waves of avian influenza A(H7N9) virus circulation in the mainland of the People’s Republic of China. BMC Infect Dis.

[20] Arai Y (2016). Novel Polymerase Gene Mutations for Human Adaptation in Clinical Isolates of Avian H5N1 Influenza Viruses. PLoS Pathog..

[21] Hatta M (2007). Growth of H5N1 influenza A viruses in the upper respiratory tracts of mice. PLoS Pathog..

[22] Taubenberger JK (2005). Characterization of the 1918 influenza virus polymerase genes. Nature..

[23] Munster VJ (2007). The molecular basis of the pathogenicity of the Dutch highly pathogenic human influenza A H7N7 viruses. J Infect Dis..

[24] Peiris JSM (2009). Innate immune responses to influenza A H5N1: friend or foe?. Trends Immunol..

[25] Long JS (2013). The effect of the PB2 mutation 627K on highly pathogenic H5N1 avian influenza virus is dependent on the virus lineage. J Virol..

[26] Mok KP (2009). Viral genetic determinants of H5N1 influenza viruses that contribute to cytokine dysregulation. J Infect Dis..

[27] Wang C, Lee HH, Yang ZF, Mok CK, Zhang Z (2016). PB2-Q591K Mutation Determines the Pathogenicity of Avian H9N2 Influenza Viruses for Mammalian Species. PLoS One..

[28] Yamada S (2011). Biological and structural characterization of a host-adapting amino acid in influenza virus. PLoS Pathog..

[29] Li Z (2005). Molecular basis of replication of duck H5N1 influenza viruses in a mammalian mouse model. J Virol..

[30] Mehle A (2009). Adaptive strategies of the influenza virus polymerase for replication in humans. Proc Natl Acad Sci USA.

[31] Yamada Shinya (2010). Biological and Structural Characterization of a Host-Adapting Amino Acid in Influenza Virus. PLoS Pathog..

[32] de Jong MD (2006). Fatal outcome of human influenza A (H5N1) is associated with high viral load and hypercytokinemia. Nat Med..

